# Associations of road traffic noise and its frequency spectrum with prevalent depression in Taichung, Taiwan

**DOI:** 10.3389/fpubh.2023.1116345

**Published:** 2023-01-27

**Authors:** Jia-Yi Lin, Wan-Ju Cheng, Chang-Fu Wu, Ta-Yuan Chang

**Affiliations:** ^1^Department of Occupational Safety and Health, College of Public Health, China Medical University, Taichung, Taiwan; ^2^Department of Public Health, College of Public Health, China Medical University, Taichung, Taiwan; ^3^Department of Psychiatry, China Medical University Hospital, Taichung, Taiwan; ^4^National Center for Geriatrics and Welfare Research, National Health Research Institutes, Miaoli, Taiwan; ^5^Institute of Environmental and Occupational Health Sciences, College of Public Health, National Taiwan University, Taipei, Taiwan

**Keywords:** cross-sectional study, depression, final particles, noise spectrum, prevalence, road traffic noise

## Abstract

**Introduction:**

Exposure to road traffic noise has been reported to be associated with depression in many epidemiological studies, but the association between noise frequency spectrum and depression remains unclear. This community-based study investigated the associations between road traffic noise exposure and its frequency components with prevalent depression.

**Methods:**

A total of 3,191 residents living in Taichung who participated in the Taiwan Biobank between 2010 and 2017, were included as study participants. The land-use regression models were used to evaluate individual annual average values of A-weighted equivalent sound level over 24 h (L_eq,24h_) and particulate matter with an aerodynamic diameter <2.5 μm (PM_2.5_) using the geographic information system. Multiple logistic regression was applied to estimate the odds ratios (ORs) for depression after adjusting for potential risk factors and PM_2.5_.

**Results:**

An interquartile range increase in L_eq,24h_ at full frequency (4.7 dBA), 1,000 Hz (5.2 dB), and 2,000 Hz (4.8 dB) was significantly associated with an elevated risk for depression with ORs of 1.62 (95% confidence interval [CI]: 1.03, 2.55), 1.58 (95% CI: 1.05, 2.37), and 1.58 (95% CI:1.03, 2.43), respectively, by controlling for PM_2.5_. The high-exposure group (≥3rd quartile median of noise levels) at full frequency, 1,000 Hz, and 2,000 Hz had an increased risk for depression with ORs of 2.65 (95% CI: 1.16–6.05), 2.47 (95% CI: 1.07–5.70), and 2.60 (95% CI: 1.10–6.12), respectively, compared with the reference group (<1st quartile of noise levels) after adjustment for PM_2.5_. Significant exposure-response trends were observed between the prevalent depression and noise exposure by quartiles at full frequency, 1,000 Hz, and 2,000 Hz (all *p* < 0.05).

**Conclusion:**

Exposure to road traffic noise may be associated with an increased prevalence of depression, particularly at 1,000 and 2,000 Hz.

## 1. Introduction

Mental disorders are a major global health concern. According to a World Health Organization (WHO) report in 2015, over one-third of the global population suffers from mental disorders annually, and the most common symptoms are depression and anxiety (1). The global burden of mental disorders continues to increase, causing considerable social and economic loss. Mental health is affected by environmental exposure and individual factors, such as genes, demographic characteristics, lifestyle, and socioeconomic status (2). The World Health Organization reports that the global population living with common mental disorders is estimated to be 4%, including 322 million people with depression (3).

Noise is defined as an uncomfortable or unwanted sound that can cause physical damage or psychological harm through various biological mechanisms (4). Exposure to noise activates the acoustic nerve to disturb the related structures in the central nervous system, such as the hypothalamic-pituitary-adrenal (HPA) axis, which is regarded as an endogenous pathway between noise and depression (5, 6). In addition, exposure to road traffic noise may affect the central nervous system and brain to increase the risk of neuropsychiatric disorders, including depressive symptoms, anxiety disorder, impaired cognitive function, dementia, and stroke (7). Noise also can cause annoyance or other negative emotions to induce psychophysiological stress responses that are related to depression (6, 8).

Many epidemiological studies have found an association between exposure to road traffic noise and depressive symptoms (9, 10). A longitudinal study reported that one A-weighted decibel (dBA) increase in daytime was significantly associated with the elevated risk on emergency hospital admissions of depression (11). A 5-year follow-up study observed that residents exposed to a 24-h noise level >55 dBA had a significantly higher risk of depressive symptoms compared with those exposed to ≤ 55 dBA (10). A case-control study revealed that inhabitants exposed to road traffic noise at a 24-h noise level ≥70 dBA had a significantly higher risk of depression (12). Subjects exposed to a road traffic noise level ≥70 dBA in day-evening-night level (Lden) showed a significantly increased prevalence of depression mood compared with those exposed to 45–54 dBA in a cross-sectional study (9). Although two meta-analysis studies concluded the low-quality evidence of road traffic noise exposure associated with medication use and interview measures of depression (13, 14), the latest review observed a marginal but not signficant elevated risk for depression per 10 dBA in Lden (15).

Different noise frequencies may be associated with the varying effects on health. Environmental noise frequencies of 63, 125, and 1,000 Hz were significantly associated with the prevalent hypertension among residents (16). Two cross-sectional studies reported the association between exposure to low-frequency noise at 10–250 Hz with annoyance (17, 18). The methods used to control the different noise levels rely on their frequency components (i.e., noise insulation for high frequencies and sound absorption for low frequencies). However, the association between frequency spectrum of road traffic noise and depression remains unclear.

In addition to road traffic noise exposure, traffic-related particulate pollutants may be associated with the depression. Previous studies have reported an association between exposure to particulate matter with an aerodynamic diameter of <2.5 μm (PM_2.5_) and an increased risk of depression (19–21). A cross-sectional study had found the association between one interquartile range (IQR) increase in PM_2.5_ levels (0.83 μg/m^3^) and an elevated prevalence of psychological distress after adjusted for road traffic noise (22), but the relationship between frequency components and depression were not investigated. To the best of our knowledge, no study has been performed to elucidate the association between noise frequency spectrum and depression. Therefore, two hypotheses were determined in the present study: (i) exposure to road traffic noise was associated with the increased risk of prevalent depression independently excluding the confounding effect of PM_2.5_; (ii) such association between road traffic noise exposure and prevalent depression was higher at low-to-medium frequencies than that at other frequency components. This study aimed to investigate the associations between road traffic noise exposure and its frequency components and the prevalence of depression after adjustment for PM_2.5_ in Taichung, Taiwan.

## 2. Materials and methods

### 2.1. Study population

This community-based cross-sectional study relied on the Taiwan Biobank database, which is a national project to systematically collect information from 125,221 inhabitants aged 30–70 years in the Taiwanese population through 29 recruitment centers, including their environmental factors, lifestyles, biomarkers, and clinical medical examinations from 2006 to 2019 in Taiwan (23, 24). Because the noise exposure was available to predict retrospectively from 2010 in Taichung city, only 3,201 residents living in Taichung city were included from the Taiwan Biobank. Nine subjects who did not provide completed questionnaire information and one participant who entered the database in 2009 were excluded. Finally, the total number of study participants was 3,191 adults (1,597 men and 1,594 women) who lived in Taichung city and participated in the Taiwan Biobank from 2010 to 2019.

The study protocol was reviewed and approved by the Central Regional Research Ethics Committee of China Medical University, Taichung, Taiwan (protocol number: CRREC-108–006). Each subject provided the written informed consent.

### 2.2. Definition of depression cases

A self-administered questionnaire released by the Taiwan Biobank was used to obtain individual data on potential risk factors for depression. These factors included age, sex, height, weight, lifestyles (such as cigarette smoking, alcohol consumption, and regular exercise), and a family history of depression. Participants were regarded as having depression if an individual answered the question: “Have you been diagnosed with depression by a physician?” In addition, participants were defined as having a family history if they answered the question, “Has your mother/father been diagnosed with depression by a physician?” at the baseline survey.

### 2.3. Exposure assessments

Based on participants' residency, we estimated the annual levels of road traffic noise and PM_2.5_ at the urban district level in Taichung city by the land-use regression method. The land-use regression (LUR) models of road traffic noise (25) and PM_2.5_ (26) established in previous studies were used to estimate individual exposure levels retrospectively when participants joined the Taiwan Biobank at baseline. The LUR model-explained variance (R^2^) of the road traffic noise for the full frequency was 83%, with the highest R^2^ of 0.88 at 250 Hz and the lowest R^2^ of 0.67 at 31.5 Hz. The precision for the full frequency was 2.09 dBA with the highest precision (2.59 dB) at 31.5 Hz and the lowest precision (1.89 dB) at 250 Hz. The accuracy for full frequency was 4.4 dBA with the highest accuracy of 6.9 dB at 1,000 Hz and the lowest accuracy of 3.6 dB at 250 Hz (25). The LUR model explained variance (R^2^) for PM_2.5_ was 0.53 with a precision of 10.2 μg/m^3^ and an accuracy of 103.9 μg/m^3^ (26). Since individuals had entered the Taiwan Biobank at different periods, the annual means of road traffic noise and PM_2.5_ exposures were adjusted for the difference between annual averages at six noise and five air-quality monitoring stations, which were setup by the Taiwan Environmental Protection Agency.

A geographic information system (ArcGIS 10.3, ESRI, Redlands, California, United States of America) was applied to integrate the parameters of land-use types, road area, road length, population numbers, and the major emission sources at different buffers to estimate the annual means of road traffic noise and PM_2.5_ for each participant between 2010 and 2019.

Based on the environmental exposure assessments, the participant were divided into four exposure groups by quartile (i.e., <1st quartile, 1st-2nd quartile, 2nd-3rd quartile, and ≥3rd quartile) in order to have the same number of subjects in each group for different frequency comparisons and tests of exposure-response trends. The median exposure level of PM_2.5_ was 33.0 μg/m^3^. In addition, the per 1-IQR increase in continuous noise variables was applied to determine the association with depression among residents in Taichung.

### 2.4. Statistical analysis

The Kolmogorov-Smirnow test was conducted to exam the normality of continuous variables for studying the association between road traffic noise exposure and prevalent depression because of sample sizes were >50. Univariate comparisons were performed using The Wilcoxon rank-sum test and the chi-square test were applied to perform univariate comparison for continuous variables and categorical variables, respectively. Spearman's correlation coefficients were calculated to exam the correlation between the road traffic noise and PM_2.5_. Logistic regression models were used to estimate ORs and 95% CIs for investigating the association between noise exposure and depression. Change-in-estimate was provided to select co-variables by trial and error for the multiple regression (27), and risk factors in multiple regression, which have an increased effect >3%, were selected to enter the models.

Single exposure variables of 24-h road traffic noise and its frequency components were built as Model 1 to estimate the risk of prevalent depression. All possible risk factors (such as age, sex, body mass index, diastolic and systolic blood pressure, alcohol consumption, betel nut chewing habits, cigarette smoking, current employment, regular exercise within the past 3 months, marital status, family history of depression, education level, monthly self-income, and monthly family income) were added to Model 1 to determine a 3% increase in the ORs of the exposure variable until no more variables exceeded this criterion. Regular exercise within 3 months, cigarette smoking, and monthly personal income were added to Model 2. Three variables to present biological plausibility, namely age, sex, and body mass index, as well as related risk factors of alcohol drinking (28), marital status (29), and a family history of depression (30) were combined with Model 2 to generate Model 3. Finally, PM_2.5_ levels were added to Model 3, accounting for the interaction to create the final model (i.e., Model 4). All analyses were conducted using the SAS standard package for Windows version 9.4 (SAS Institute Inc., Cary, North Carolina, USA). The significance level was set at a *p* < 0.05 for all statistical tests.

## 3. Results

Table 1 shows the demographic characteristics of the study participants in the Taiwan Biobank from 2010 to 2019. Significant differences were identified between depressive and non-depressive groups in body mass index, sex, marital status, currently employed, monthly self-income, and family history of depression (all *P* < 0.05).

**Table 1:** Demographic characteristics of study participants in Taiwan Biobank from 2010 to 2019.

**Characteristics**	**Depression (*n =* 102)**	**Non-depression** ** (*n =* 3,089)**	***P*-value**
Age (year), mean (SD)	48.51 (11.37)	47.96 (11.10)	0.600^a^
BMI (kg/m^2^), mean (SD)	23.78 (3.97)	24.39 (3.79)	0.046^a^
Sex, male (%)	40 (39.22)	1,557 (50.40)	0.026^b^
Education level, >12 years (%)	54 (52.94)	1,291 (41.79)	0.289^b^
Marriage, yes (%)	17 (16.67)	415 (13.43)	<0.001^b^
Divorced or widow, yes (%)	23 (22.55)	288 (9.32)	<0.001^b^
Currently employed, yes (%)	23 (22.55)	849 (63.36)	0.003^b^
Monthly self-income, >30,000 NTD (%)	19 (18.63)	847 (27.48)	0.049^b^
Monthly family income, >80,000 NTD (%)	18 (17.65)	555 (17.97)	0.934^b^
Cigarette smoking, yes (%)	19 (18.63)	381 (12.33)	0.056^b^
Alcohol consumption, yes (%)	12 (11.76)	266 (8.61)	0.268^b^
Betel-nut chewing, yes (%)	3 (2.94)	88 (2.85)	0.767^b^
Regular exercise within past 3 months, yes (%)	37 (36.27)	1,197 (38.75)	0.612^b^
Family history of depression, yes (%)	17 (16.67)	179 (5.79)	<0.001^b^

BMI, body mass index; NTD, New Taiwan dollar; SD, standard deviation.

a Wilcoxon rank-sum test for significant differences (P < 0.05) between depressive and non-depressive subjects.

b Chi-square test for significant differences (P < 0.05) between depressive and non-depressive subjects.

Table 2 presents the annual mean values of the 24-h road traffic noise and PM_2.5_. The annual mean of L_eq,24h_ for full frequency was 68.12 ± 3.74 dBA, with the highest value of 64.47 ± 4.24 dB at 1,000 Hz. The annual mean concentration of PM_2.5_ was 32.39 ± 5.38 μg/m^3^ and ranged from 17.68 to 46.98 μg/m^3^.

**Table 2:** Distributions of annual 24-h road traffic noise and fine particles.

**Exposure Level**	**Mean ±SD**	**Median**	**Range**	**Q1, Q3**	**IQR**
L_eq,24h_ (dBA)	68.12 ± 3.74	69.26	57.39–72.71	66.69, 71.42	4.73
31.5 Hz (dB)	27.95 ± 1.43	27.90	25.38–31.59	26.86, 29.45	2.59
63 Hz (dB)	42.36 ± 3.19	43.08	29.39–54.65	41.41, 44.63	3.22
125 Hz (dB)	51.13 ± 3.09	51.50	37.92–57.32	49.15, 52.87	3.72
250 Hz (dB)	55.43 ± 3.49	56.17	44.13–61.33	53.08, 57.59	4.51
500 Hz (dB)	60.12 ± 3.08	60.86	50.43–65.46	58.26, 62.25	3.99
1,000 Hz (dB)	64.47 ±4.24	65.49	48.97–71.15	62.83, 68.00	5.17
2,000 Hz (dB)	62.22 ± 4.06	63.49	48.90–67.28	60.89, 65.70	4.81
4,000 Hz (dB)	57.64 ± 2.99	58.39	47.32–61.20	56.72, 59.58	2.86
8,000 Hz (dB)	56.20 ± 2.46	56.52	42.91–63.31	54.78, 58.04	3.26
PM_2.5_ (μg/m^3^)	32.39 ± 5.38	33.00	17.68–46.98	28.41, 36.60	8.19

dB, decibel; dBA, A-weighted decibel; IQR, Interquartile range; SD, Standard deviation.

The correlations between the annual 24-h road traffic noise and PM_2.5_ are shown in Table 3. The PM_2.5_ level was significantly correlated with 24-h road traffic noise levels at full (correlation coefficient = 0.339) and spectrum frequencies (all *P* < 0.001), which was observed with the highest correlation (coefficients = 0.636) at 31.5 Hz and with the lowest correlation (coefficients = 0.173) at 31.5 and 250 Hz, respectively.

**Table 3:** Correlations between the annual 24 h road traffic noise and fine particles.

**Exposure variables**	**PM**_**2.5**_ **(**μ**g/m**^**3**^**)**	

	**r**	***P*** **-value**
L_eq,24h_ (dBA)	0.34	<0.001
31.5 Hz (dB)	0.64	<0.001
63 Hz (dB)	0.59	<0.001
125 Hz (dB)	0.31	<0.001
250 Hz (dB)	0.17	<0.001
500 Hz (dB)	0.45	<0.001
1,000 Hz (dB)	0.29	<0.001
2,000 Hz (dB)	0.34	<0.001
4,000 Hz (dB)	0.53	<0.001
8,000 Hz (dB)	0.51	<0.001

dB, decibel; dBA, A-weighted decibel; r, Spearman correlation coefficient.

The associations between an interquartile range (IQR) increase in annual 24-h road traffic noise and prevalent depression are shown in Table 4. An IQR increase in full frequency (4.7 dBA), spectrum frequency at 1,000 Hz (5.2 dB), and frequency component at 2,000 Hz (4.8 dB) were significantly associated with an increased risk of depression (OR = 1.62, 95% CI: 1.03–2.55; OR = 1.58, 95% CI: 1.05–2.37; OR = 1.58, 95% CI: 1.03–2.43) after adjusting for potential risk factors and PM_2.5_.

**Table 4:** Associations between one interquartile-range increase in annual 24-h road traffic noise and prevalent depression.

**Variables**	**Model 1**		**Model 2**		**Model 3**		**Model 4**	

	**Crude OR** **(95% CI)**	***P*** **-value**	**Adjusted OR** **(95% CI)**	***P*** **-value**	**Adjusted OR** **(95% CI)**	***P*** **-value**	**Adjusted OR** **(95% CI)**	***P*** **-value**
**Noise level**								
L_eq,24h_ (4.7 dBA)	1.32 (0.99–1.75)	0.056	1.69 (1.10–2.58)	0.016	1.61 (1.05–2.48)	0.029	1.62 (1.03–2.55)	0.037
**Frequency components**								
31.5 Hz (2.6 dB)	1.09 (0.76–1.56)	0.644	1.52 (0.92–2.50)	0.099	1.34 (0.80–2.25)	0.268	1.36 (0.70–2.62)	0.363
63 Hz (3.2 dB)	0.99 (0.81–1.20)	0.911	0.99 (0.76–1.28)	0.931	0.94 (0.73–1.22)	0.639	0.87 (0.66–1.16)	0.344
125 Hz (3.7 dB)	1.10 (0.86–1.40)	0.466	1.09 (0.79–1.50)	0.620	1.04 (0.75–1.43)	0.827	1.01 (0.72–1.41)	0.951
250 Hz (4.5 dB)	1.23 (0.93–1.63)	0.141	1.54 (1.03–2.32)	0.036	1.49 (0.99–2.24)	0.059	1.47 (0.98–2.22)	0.066
500 Hz (4.0 dB)	1.22 (0.92–1.61)	0.170	1.52 (1.02–2.29)	0.042	1.48 (0.98–2.24)	0.065	1.48 (0.94–2.32)	0.088
1,000 Hz (5.2 dB)	1.34 (1.02–1.75)	0.036	1.64 (1.11–2.42)	0.013	1.58 (1.06–2.35)	0.025	1.58 (1.05–2.37)	0.029
2,000 Hz (4.8 dB)	1.31 (1.00–1.71)	0.049	1.65 (1.10–2.47)	0.016	1.58 (1.05–2.38)	0.029	1.58 (1.03–2.43)	0.036
4,000 Hz (2.9 dB)	1.21 (0.96–1.51)	0.101	1.43 (1.01–2.02)	0.043	1.38 (0.97–1.94)	0.071	1.38 (0.94–2.01)	0.099
8,000 Hz (3.3 dB)	1.04 (0.79–1.37)	0.762	1.05 (0.73–1.51)	0.780	1.00 (0.70–1.44)	0.989	0.96 (0.66–1.39)	0.823

CI, Confidence interval; dB, decibel; dBA, A-weighted decibel; OR, Odds ratio.

Model 1, single exposure variable; Model 2, Model 1 with adjustment for regular exercise within 3 months, cigarette smoking, and monthly self-income; Model 3, Model 2 with adjustment for age, sex, BMI, drinking, married vs. unmarried and married vs. divorced, and family history; Model 4, Model 3 with adjustment for PM_2.5_.

Table 5 presents the associations between dichotomous noise exposure groups (by quartile) and prevalent depression. The OR for prevalent depression was significantly higher in high-exposure group (≥3rd quartile, Q3) at the full frequency and spectrum frequencies of 1,000 and 2,000 Hz compared with the reference group (< 1st quartile, Q1) after controlling for potential risk factors and PM_2.5_. Participants exposed to ≥71.4 dBA at full frequency, those exposed to ≥68.0 dB at 1,000 Hz, and those exposed to ≥65.7 dB at 2,000 Hz had significantly higher risks of 2.65 (95% CI: 1.16–6.05), 2.47 (95% CI: 1.07–5.70), and 2.60 (95% CI: 1.10–6.12) than the reference groups, respectively. Significant exposure-response trends were identified between the prevalent depression and the stratum of noise exposure at full frequency (OR = 1.40, 95% CI: 1.09–1.79, *p* = 0.009), 1,000 Hz (OR = 1.37, 95% CI: 1.06–1.77, *p* = 0.015), and 2,000 Hz (OR = 1.42, 95% CI: 1.09–1.84, *p* = 0.009).

**Table 5:** Associations between different 24-h road traffic noise exposure groups (by quartile) and prevalent depression.

**Variables**	**Model 3**		**Model 4**		**Trend**

	**Adjusted OR** **(95% CI)**	***P*** **-value**	**Adjusted OR** **(95% CI)**	***P*** **-value**	***P*** **-value**
**Noise level, L**_eq,24h_					
66.7–69.3 vs. < 66.7 dBA	1.03 (0.42–2.54)	0.949	1.06 (0.42–2.71)	0.902	**0.009**
69.3–71.4 vs. < 66.7 dBA	1.33 (0.58–3.05)	0.503	1.37 (0.57–3.26)	0.479	
≥71.4 vs. < 66.7 dBA	2.55 (1.20–5.42)	0.015	**2.65 (1.16–6.05)**	**0.021**	
**Frequency components**					
31.5 Hz, 26.9–27.9 vs. < 26.9 dB	0.82 (0.36–1.91)	0.653	0.84 (0.35–2.02)	0.688	0.201
31.5 Hz, 27.9–29.5 vs. < 26.9 dB	0.91 (0.41–2.02)	0.822	0.93 (0.39–2.23)	0.870	
31.5 Hz, ≥29.5 vs. < 26.9 dB	1.56 (0.74–3.32)	0.247	1.61 (0.62–4.17)	0.329	
63 Hz, 41.4–43.1 vs. < 41.4 dB	0.52 (0.23–1.21)	0.128	0.46 (0.18–1.17)	0.102	0.278
63 Hz, 43.1–44.6 vs. < 41.4 dB	0.45 (0.17–1.17)	0.100	0.39 (0.14–1.12)	0.080	
63 Hz, ≥44.6 vs. < 41.4 dB	1.30 (0.67–2.52)	0.433	1.10 (0.46–2.59)	0.833	
125 Hz, 49.2–51.5 vs. < 49.2 dB	0.62 (0.27–1.44)	0.265	0.46 (0.18–1.15)	0.096	0.376
125 Hz, 51.5–52.9 vs. < 49.2 dB	0.79 (0.36–1.71)	0.547	0.51 (0.20–1.31)	0.162	
125 Hz, ≥52.9 vs. < 49.2 dB	1.29 (0.65–2.57)	0.465	1.07 (0.51–2.21)	0.864	
250 Hz, 53.1–56.2 vs. < 53.1 dB	1.22 (0.52–2.84)	0.644	1.19 (0.49–2.87)	0.699	0.141
250 Hz, 56.2–57.6 vs. < 53.1 dB	1.64 (0.75–3.58)	0.216	1.57 (0.66–3.75)	0.307	
250 Hz, ≥57.6 vs. < 53.1 dB	1.70 (0.76–3.77)	0.194	1.67 (0.74–3.76)	0.215	
500 Hz, 58.3–60.9 vs. < 58.3 dB	1.21 (0.50–2.93)	0.666	1.26 (0.51–3.08)	0.615	0.059
500 Hz, 60.9–62.3 vs. < 58.3 dB	2.20 (1.01–4.79)	0.047	2.39 (1.01–5.66)	0.047	
500 Hz, ≥62.3 vs. < 58.3 dB	1.84 (0.78–4.34)	0.162	2.01 (0.79–5.16)	0.144	
1,000 Hz, 62.7–65.5 vs. < 62.7 dB	1.16 (0.48–2.79)	0.749	1.14 (0.45–2.87)	0.780	**0.015**
1,000 Hz, 65.5–68.0 vs. < 62.7 dB	1.32 (0.56–3.10)	0.521	1.30 (0.53–3.19)	0.561	
1,000 Hz, ≥68.0 vs. < 62.7 dB	2.51 (1.14–5.50)	0.022	**2.47 (1.07–5.70)**	**0.033**	
2,000 Hz, 60.7–63.5 vs. < 60.7 dB	0.96 (0.39–2.37)	0.936	0.98 (0.38–2.51)	0.963	**0.009**
2,000 Hz, 63.5–65.7 vs. < 60.7 dB	1.29 (0.55–2.98)	0.559	1.31 (0.54–3.18)	0.557	
2,000 Hz, ≥65.7 vs. < 60.7 dB	2.55 (1.16–5.59)	0.020	**2.60 (1.10–6.12)**	**0.029**	
4,000 Hz, 56.7–58.4 vs. < 56.7 dB	0.95 (0.41–2.20)	0.899	0.99 (0.42–2.38)	0.989	0.103
4,000 Hz, 58.4–59.6 vs. < 56.7 dB	1.09 (0.49–2.42)	0.836	1.17 (0.50–2.76)	0.719	
4,000 Hz, ≥59.6 vs. < 56.7 dB	1.82 (0.85–3.88)	0.123	2.09 (0.80–5.50)	0.134	
8,000 Hz, 54.8–56.5 vs. < 54.8 dB	0.58 (0.24–1.39)	0.219	0.53 (0.21–1.37)	0.189	0.202
8,000 Hz, 56.5–58.0 vs. < 54.8 dB	0.57 (0.25–1.33)	0.194	0.53 (0.22–1.31)	0.169	
8,000 Hz, ≥58.0 vs. < 54.8 dB	1.50 (0.77–2.94)	0.233	1.35 (0.60–3.04)	0.475	

CI, Confidence interval; dB, decibel; dBA, A-weighted decibel; OR, Odds ratio.

Model 3, adjustment for age, sex, BMI, monthly self-income, married vs. unmarried and married vs. divorced, cigarette smoking, alcohol drinking, regular exercise within 3 months, and family history; Model 4, Model 3 with adjustment for PM_2.5_ levels. The bold values indicate the significant results (*p* < 0.05) in regression models.

We also conducted the analyses of interaction effects between road traffic noise (including different frequency components) and PM_2.5_, but no significant interaction effects were observed (all *p* > 0.05) after adjusting for age, sex, BMI, cigarette smoking, alcohol drinking, married status, monthly self-income, regular exercise within 3 months, and family history.

## 4. Discussion

### 4.1. Main findings

This study found that exposure to 24-h road traffic noise was significantly associated with the prevalent risk of depression after adjusting for PM_2.5_. Participants exposed to 24-h road traffic noise ≥71.4 dBA had a significant higher risk of depression than those exposed to <66.7 dBA, and an IQR (4.7 dBA) increase in full frequency was significantly associated with the prevalent depression. These findings are similar to those reported in past studies. A linear exposure-response relationship was found in road traffic noise with an OR of 1.17 (95% CI: 1.10–1.25) for 24-h continuous sound levels ≥70 dB (12). A German prospective cohort study pointed out that when compared with the ≤ 55 dBA category, the incidence of depressive symptoms was significantly higher than in those with an exposure >55 dBA (RR=1.29, 95% CI: 1.03–1.62) category after adjusting for traffic proximity (10). However, we observed a stronger association between 24-h road traffic noise exposure and depression compared with those findings after controlling for the confounders of PM_2.5_. Exposure to one IQR (16.7 μg/m^3^) of PM_2.5_ was found to be associated with self-reported psychological distress (OR=1.09, 95% CI: 1.07–1.12), hypnotic and sedative use (OR=1.04, 95% CI: 1.00–1.09), and antidepressant treatments (OR=1.01, 95% CI: 1.00–1.03) after adjusting for road traffic noise (22). Therefore, both road traffic noise and PM_2.5_ should be considered to estimate the unbiased impacts on mental health.

The possible biological mechanism of road traffic noise exposure in depression is that noise may activate the central nervous system of emotional processing as a threat to homeostasis (31). There are two main allostatic regulatory systems for stress responses: the HPA axis and the sympathetic–adrenal–medullary axis (32). Chronic exposure to stress may affect the HPA axis to generate metabolic changes that pose the impaired immune function, diabetes, depressive symptoms, and cognitive disturbances (33). An animal experimental study showed that rats exposed to noise produced more free radicals, which might increase superoxide dismutase activity (34), consequently causing systemic inflammation and oxidative stress. These responses lead to the destruction of the nervous system and melancholic behaviors (35).

This is the first study to determine the association between the frequency spectrum of road traffic noise and the prevalence of depression. Residents exposed to mid-high frequencies of road traffic noise (i.e., 1,000 and 2,000 Hz) were significantly associated with an elevated risk of depression to present exposure-response trends. Another mechanism underlying the association between noise and depression is chronic physical illness. The prevalent depression was found to be higher among diabetic patients (20%) than among asthmatics (12%) and healthy individuals (4%) in a comparative study (36). Furthermore, the hypertensive and diabetic subjects had the higher prevalent depression than the general population in Peruvian (37). Mid-to-high-frequency noise exposure has an adverse effect on insulin control and leads to a rise in blood sugar, which may cause type 2 diabetes mellitus between 1,000 and 2,000 Hz (38). Besides the insulin control, noise exposure may cause inflammatory pathways and oxidative stress to drive the adverse health effects on brain (7). In addition, exposure to road traffic noise at 1,000 Hz has been found to have a significant and positive relationship with prevalent hypertension (16) that is a risk factor of stroke. Low-frequency noise at 10–250 Hz was found to be associated with annoyance (17, 18), but this study did not observe significant associations between prevalent depression and noise exposure at low-frequency components. These evidences indicate that people may have higher perception to noise at frequencies higher than 1,000 Hz. Therefore, it was inferred that exposure to frequency components of 1,000 and 2,000 Hz might affect the prevalence of depression in chronic diseases.

The present study did not show a significant association between PM_2.5_ exposure and prevalent depression. In addition, further analyses of interaction effects between noise frequency components and PM_2.5_ did not find any significant interactions. However, a cross-sectional study had observed the association between one IRQ increase in PM_2.5_ and prevalence of psychological distress after adjusting for road traffic noise (22). The possible reasons for this inconsistence may be that psychological distress is a symptom with sentiment which may change over a short period of time (39).

### 4.2. Strengths and limitations

The major advantage in this study was the application of predictive models with the high predictive ability (adjusted R^2^: 0.7–0.8) for both 24-h road traffic noise and their frequency components (25), and a moderate predictive ability (adjusted R^2^: 0.5) for PM_2.5_ (26). These models provide better exposure assessments for residents living in Taichung, Taiwan than traditional approaches used in previous studies by either the closest monitoring station (40) or inverse distance relationship (41) to investigate the associations between road traffic noise and air pollutants with adverse health effects. Another advantage is the application of the Taiwan Biobank database, which is systematic sampling data that can represent the general population in Taichung. Furthermore, the present study is the first to determine the association between noise spectrum and prevalence of depression.

However, the present study has some limitations. First, we could not build a causality between depression and noise exposure because of the inherent restriction of temporality in the cross-sectional study design. Second, depression was assessed using one question in the survey, and no medical records were available to confirm the time of diagnosis and remission. It would be better to use a standardized scale for assessing depressive symptoms because many people with depression will never be diagnosed by a physician as they will not attend to the doctor. Third, the land-use regression method was a space-time geostatistical algorithm based on resident address, which limited the precise measurements of individual exposure levels. The non-differential misclassification of exposure for all subjects might bias the effect estimate to generate the null value of 1.0, but we still observed the significant associations between exposure to road traffic noise and specific noise spectrum and the prevalent depression. Fourth, temperature was not adjusted in our model, although it was reported to be associated with an increased risk of incident depression (RR=1.31, 95% CI: 1.09–1.56) at a daily mean temperature of 16.4°C (42). Fifth, the lack of data on sleep quality information might lead to overestimation of noise exposure effects on depression. Noise is a strong risk factor for depression because it has been found to cause poor sleep quality (43). Finally, work-related job stress, which was a confounder to be adjusted for in the data analysis, was not measured in this study (44).

## 5. Conclusions

In summary, we found a significant association between 24-h road traffic noise exposure and an increased risk of prevalent depression. The mid-to-high-frequency components at 1,000 and 2,000 Hz were found to be related to the prevalent depression, providing a possible link between noise exposure and mental health. We recommend that future studies are performed using a longitudinal design to confirm these findings.

## Data availability statement

The dataset of Taiwan Biobank is available for registration and requirement. Requests to access these datasets should be directed to Taiwan Biobank, biobank@gate.sinica.edu.tw.

## Ethics statement

The studies involving human participants were reviewed and approved by Central Regional Research Ethics Committee of China Medical University, Taichung, Taiwan (protocol number: CRREC-108–006). The patients/participants provided their written informed consent to participate in this study.

## Author contributions

J-YL and T-YC conceived and designed the study and performed data analyses. J-YL, W-JC, C-FW, and T-YC collected and assembled the data. J-YL, W-JC, and T-YC wrote the manuscript. C-FW made critical revision of the manuscript for key intellectual content. T-YC handled funding and supervision. All authors contributed to the article and approved the submitted version.
